# Motor Fatigue Measurement by Distance-Induced Slow Down of Walking Speed in Multiple Sclerosis

**DOI:** 10.1371/journal.pone.0034744

**Published:** 2012-04-13

**Authors:** Rémy Phan-Ba, Philippe Calay, Patrick Grodent, Gael Delrue, Emilie Lommers, Valérie Delvaux, Gustave Moonen, Shibeshih Belachew

**Affiliations:** 1 MYelin Disorders REseArch teaM (MYDREAM), Liège, Belgium; 2 Department of Neurology, University and C.H.U. of Liège, Liège, Belgium; 3 Department of Physical Medicine and Rehabilitation, University and C.H.U. of Liège, Liège, Belgium; University Hospital of Heidelberg, Germany

## Abstract

**Background and rationale:**

Motor fatigue and ambulation impairment are prominent clinical features of people with multiple sclerosis (pMS). We hypothesized that a multimodal and comparative assessment of walking speed on short and long distance would allow a better delineation and quantification of gait fatigability in pMS. Our objectives were to compare 4 walking paradigms: the timed 25-foot walk (T25FW), a corrected version of the T25FW with dynamic start (T25FW^+^), the timed 100-meter walk (T100MW) and the timed 500-meter walk (T500MW).

**Methods:**

Thirty controls and 81 pMS performed the 4 walking tests in a single study visit.

**Results:**

The 4 walking tests were performed with a slower WS in pMS compared to controls even in subgroups with minimal disability. The finishing speed of the last 100-meter of the T500MW was the slowest measurable WS whereas the T25FW^+^ provided the fastest measurable WS. The ratio between such slowest and fastest WS (Deceleration Index, DI) was significantly lower only in pMS with EDSS 4.0–6.0, a pyramidal or cerebellar functional system score reaching 3 or a maximum reported walking distance ≤4000 m.

**Conclusion:**

The motor fatigue which triggers gait deceleration over a sustained effort in pMS can be measured by the WS ratio between performances on a very short distance and the finishing pace on a longer more demanding task. The absolute walking speed is abnormal early in MS whatever the distance of effort when patients are unaware of ambulation impairment. In contrast, the DI-measured ambulation fatigability appears to take place later in the disease course.

## Introduction

Multiple sclerosis (MS) is a chronic multifocal disease of the CNS, which produces a wide range of neurological deficits. Ambulation impairment is recognized as a prominent feature of disability in MS, both by physicians and people with MS (pMS) [1]. The mechanisms underlying this locomotor impairment remain partially elusive. Besides functional system neurological deficits observed in the course of MS, it has been hypothesized that MS related motor fatigue can also impede gait performances [2]. In this context, motor fatigue is defined as the gradual decline of the maximal muscle strength during a constant mild to moderate physical exercise. Evaluation of ambulation limitation plays a central role in clinical scales [3] and composite outcome measures [4], [5], which are used in the routine clinical practice and randomized clinical trials. The quantification of gait performances in MS remains usually limited to the simple anamnestic recall of the maximum reported walking distance (MrWD) [3], the stopwatch measurement of walking speed on short distance walking tests [4], [5] through various settings and methodologies [6]–[11], and the measurement of the maximum distance performed in a given time [12]. In contrast to maximum walking distance or maximum walking time, walking speed (WS) is believed to be a more stable parameter, which is less day-to-day variable and can be extracted from various walking paradigms [13], [14]. Only few studies have investigated the behavior of pMS' performances on longer distance walking tests, with variable results and methodologies, as well as small population samples [2], [12]. Gait is a complex motor behaviour that can only be roughly disentangled by a single walking test and we previously hypothesized that a multimodal walking assessment of gait in pMS would allow a better delineation and quantification of functional gait impairment in MS [7].

Since the onset of permanent gait limitations has often been conceived as a late process in the course of the disease, ambulation performances are only taken into account beyond the score of 4.0 in the Expanded Disability Status Scale (EDSS) [3]. However, several studies have suggested that the restriction of ambulation performances occurs much earlier than previously considered [15]–[17], but the precise timing and the extent of such limitations have been scarcely investigated.

In this work, we developed a 500-meter walking test to evaluate the mean WS of pMS in a demanding distance-based effort in comparison to the conventional short distance 25-foot test in a similar “as fast as possible” paradigm. Our objectives were (i) to determine the range of performances of pMS in this long-distance walking modality, (ii) to study the deceleration of the WS over this 500-meter distance in different subsets of pMS stratified according to their global EDSS, functional system (FS) scores according to Kurtzke and MrWD below or above the 4000 m milestone. These results emphasized that deceleration over the distance of a demanding ambulation test may be a valuable tool to assess locomotor fatigability in MS.

## Methods

### Ethics Statement

The “Comité d'Ethique hospitalo-facultaire” of the CHU of Liège approved the study procedure and written informed consent was received from all participants.

### Methods

A total of 81 subjects with a diagnosis of relapsing–remitting or progressive MS according to the McDonald criteria [18] and a MrWD≥500 m, and 30 weight- and sex-matched healthy volunteers used as a control group were enrolled in the study. pMS who had an EDSS from 4.5 to 6.0 were allowed to perform the walk tests using ambulatory assistive devices in case they would usually need it to walk the distance of 500 m or more. In such conditions (n = 9), the only requirement was that they were asked to use the same device for all tests. Ankle–foot orthosis was permitted if worn from onset for all evaluations. pMS who had experienced clinically disabling MS exacerbations with or without corticosteroid treatment within the last 3 months before study enrollment were excluded. Since it was previously shown that the time of the day does not interfere with ambulation outcome performances despite changes in subjective fatigue [14], pMS were tested at random periods of the day at their most convenient time.

pMS and healthy controls performed a multimodal walking assessment that comprised 4 tests, in the following order : the Timed 25-Foot Walk Test (T25FW, performed twice), a corrected version of the T25FW with a dynamic start (T25FW^+^, performed twice [10]), the Timed 100-Meter Walk Test (T100MW [7]), and the Timed 500-Meter Walk Test (T500MW). A period of rest of 15 minutes was allowed between each test to minimize interference due to potential test-related fatigue, and all demanding physical activities (such as rehabilitation sessions) were suspended in the last 24 hours prior to the assessment. Our subjects did not report any increased sense of subjective fatigue before starting a new test, especially before the last and most demanding T500MW. A slight worsening of the absolute results due to an increased motor fatigue in the T500MW cannot be excluded but this methodological bias was identical for all subjects.

All assessments were made by a certified MS nurse (PC) or by a physical therapist in charge of patients' rehabilitation programs (PG). EDSS scores were all collected by a certified EDSS rater (RP or SB).

The MrWD was evaluated as follows: control healthy volunteers all reported a MrWD superior to 4000 m, which was considered as “unlimited”. pMS were asked whether they had the feeling that during the past 4 weeks their average walking performance had been unlimited and whether they thought they could walk for 4000 m or more without aid or rest. If they answered “yes”, they were considered to have an “unlimited” MrWD (i.e. ≥4000 m). pMS who considered themselves unable to walk 4000 m without aid or rest were asked to evaluate as accurately as possible their MrWD, which was defined as the maximum distance they thought they could walk without rest, and over which they would estimate they have a high risk of falling in case they would go on for a few meters more.

The T25FW was performed according to the published standardized instructions [4], [5]. The T25FW^+^ was also strictly following the guidelines of the T25FW [4], [5], except that the subjects were allowed to take a 3 meters run-up before the starting line [10]. This run-up was clearly demarcated on the ground. In order to minimize test-retest variability, the mean value of the two tests was used in the analysis of the T25FW and the T25FW^+^.

The T500MW was performed as 5 non-stop consecutive laps of the same path that served for the T100MW, as previously described [19], where interval times were recorded at each 100 m. The T100MW and T500MW were performed in a 3 m width corridor, devoid of obstacles. Running was prohibited. The subject was directed just behind the starting line and then instructed as follows: “I'd like you to walk this 100 (or 500) meter distance as quickly as possible, but safely. Do not slow down until after you've passed the finish line. Ready? Go.” Timing started when the lead foot crossed the starting line. The examiner could not walk along with the patient as he/she completed the task. Timing was stopped when the lead foot crossed the finish line. The examiner then recorded the subject's walking time to within 0.1 second, rounding up or down as necessary. We rounded up to the next tenth if the hundredth of a second's place was ≥.05, rounded down if the hundredth of a second's place was <.05 (eg, 55.45″ would round up to 55.5″ but 55.44″ would round down to 55.4″).

The mean walking speed (MWS) expressed in meters per second were obviously calculated by dividing 7,62 m (i.e. 25 foot), 100 m or 500 m by the time to perform the respective distances.

Comparisons between groups were made with a student t-test and comparison within group with a paired t-test. All statistical tests were applied with a two-tailed analysis and 0.05 as a level of significance and were performed using GraphPad Prism, version 4.0b for Macintosh, GraphPad Software, San Diego California USA (www.graphpad.com).

## Results

The baseline characteristics of healthy control volunteers and pMS are detailed in Table 1. The distributions of gender and weight were comparable in both groups. The MS population was well balanced between different ranges of clinical disability stratified from EDSS 0 to 2.0, 2.5 to 3.5 and 4.0 to 6.0. Sixty percent of our MS population had an unlimited walking range defined by a MrWD≥4000 m, whereas approximately 40% reported to be able to walk between 500 m and 4000 m. MS patients were also stratified according to pyramidal, cerebellar and sensitive Kurtzke FS scores (all FS≤1, FS = 2 or FS = 3, no patients had an FS>3 in one of these three systems).

**Table 1:** Baseline characteristics of people with MS and healthy control volunteers.

	pMS	Healthy controls
**Number**	81	30
**Age (years; mean ± SD)**	40.16±11.35	30.3±10.4
**Sex (female, %)**	59	70
**BMI** 1 **(mean ± SD)**	23.72±4.13	23.33±3.37
**MS type (CIS/RR/SP/PP** 2 **, %)**	10.1/61.7/14.6/13.4	n.a.
**Disease duration (years; mean ± SD)**	9.75±8.79	n.a.
**EDSS** 3 **(median; range)**	3.5 (0–6.0)	n.a.
**0–2.0 (n, %)**	30, 37	n.a.
**2.5–3.5 (n, %)**	21, 25.9	n.a.
**4.0–6.0 (n, %)**	30, 37	n.a.
**All FS** 4 **≤1 (n, %)**	21, 25.9	n.a.
**FS Pyramidal = 2, irrespective of other FS (n, %)**	15, 18.5	n.a.
**FS Cerebellar = 2, irrespective of other FS (n, %)**	18, 22.2	n.a.
**FS Sensitive = 2, irrespective of other FS (n, %)**	34, 41.9	n.a.
**FS Pyramidal = 3, irrespective of other FS (n, %)**	25, 30.9	n.a.
**FS Cerebellar = 3, irrespective of other FS (n, %)**	31, 38.3	n.a.
**FS Sensitive = 3, irrespective of other FS (n, %)**	15, 18.5	n.a.
**MrWD** 5		
**≥4000 meters (n, %)**	49, 60.5	n.a.
**≥500 meters; <4000 meters (n, %)**	32, 39.5	n.a.

1 ; Body Mass Index (kg/cm^2^);

2 : clinically isolated syndrome/relapsing-remitting/secondary progressive/primary progressive - progressive-relapsing;

3 : Expanded Disability Status Scale;

4 : Kurtzke Functionnal System Score;

5 : Maximum reported Walking Distance.

**Figure 1:**
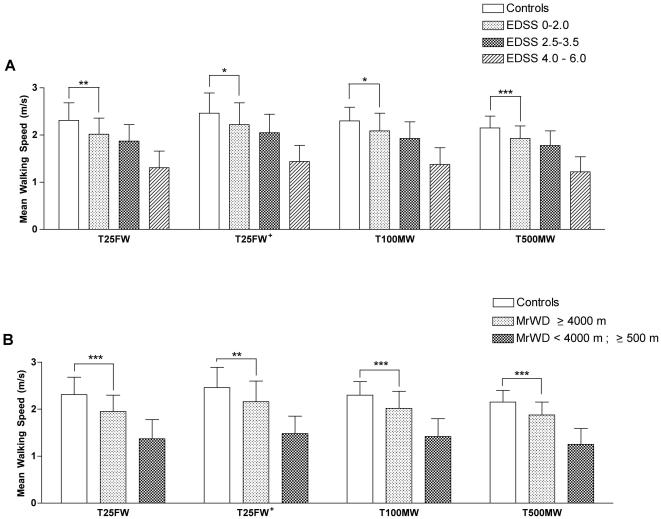
Mean walking speed (MWS) in healthy volunteers and in different subgroups of the pMS population. The same general pattern of MWS differences across the different walking paradigms is observed in every group (T25FW^+^>T100MW>T25FW>T500MW). In the 4 walking tests, the MWS was significantly slower for each subset of the pMS population compared to healthy volunteers (all p<0,0001), including pMS with a low level of disability according to their EDSS status (EDSS≤2.0, A) or an apparently unlimited MrWD (MrWD≥4000 m, B).

Mean timed performances in the 4 walking tests for healthy volunteers and for the different subgroups of pMS are presented in Table 2. For the T500MW, lap times per 100 m are also presented (Table 2). The mean walking speed (MWS) was compared between the 4 tests (Figure 1) in healthy volunteers and pMS according to their EDSS and MrWD. In healthy volunteers and in all subsets of pMS regardless of their EDSS or MrWD status, the order of calculated MWS values was T25FW^+^>T100MW>T25FW>T500MW. In all short and longer distance walking tests, the MWS was significantly lower for each subset of the pMS population compared to healthy volunteers (statistics only shown graphically in Fig. 1A and 1B for pMS with EDSS≤2.0 or an apparently unlimited MrWD≥4000 m). MWS was also significantly lower for pMS at EDSS 4.0–6.0 compared to EDSS 2.5–3.5, in the 4 walking tests (Figure 1A, p<0.001 for all comparisons). No significant difference was found between the MWS of the pMS at EDSS 0–2.0 compared to EDSS 2.5–3.5 (p = 0.1419 for T25FW, p = 0.1987 for T25FW^+^, p = 0.1178 for T100MW, and p = 0.0783 for T500MW). Finally, MWS was significantly higher for pMS with an MrWD≥4000 m compared to that of patients with an MrWD<4000 m in the 4 walking tests (Figure 1B, p<0.001 for all comparisons). When pMS were stratified according to pyramidal, cerebellar and sensitive Kurtzke FS scores, MWS data for all walking tests were very sensitive to detect significant differences between pMS with all FS≤1 and pMS with at least one FS = 2 or to detect significant differences between pMS with one FS = 2 and pMS with the same FS = 3 (Table S1).

**Table 2:** Timed performances^1^ of respective populations in the different walking tests.

	Controls (30)	pMS					
		All (81)	EDSS 0–2.0 (30)	EDSS 2.5–3.5 (21)	EDSS 4.0–6.0 (30)	MrWD≥4000 (49)	MrWD 500–4000 (32)
**T25FW** 2	3.38±0.53	4.91±2.10	3.88±0.64	4.21±0.76	6.44±2.73	4.04±0.77	6.25±2.72
**T25FW^+^** 3	3.17±0.48	4.42±1.57	3.57±0.69	3.85±0.77	5.66±1.82	3.67±0.74	5.56±1.80
**T100MW** 4	44.05±5.50	61.26±22.59	49.23±8.27	53.69±10.50	78.59±27.60	51.02±9.60	76.94±27.48
**T500MW** 5	235.28±27.80	338.32±134.23	265.25±44.89	289.50±53.66	445.56±162.97	272.28±43.49	439.44±161.43
**0–100**	45.29±5.87	63.08±22.03	50.05±8.35	55.23±10.32	81.59±24.91	51.86±9.10	80.26±24.90
**100–200**	46.97±6.92	67.15±25.55	53.14±8.16	57.96±12.27	87.59±30.57	54.45±8.37	86.59±30.52
**200–300**	47.81±5.30	67.91±26.45	53.86±8.00	58.73±10.72	88.39±32.98	55.42±8.50	87.05±32.70
**300–400**	48.14±5.83	69.36±22.03	54.18±10.25	58.87±11.34	91.89±35.63	55.48±9.37	90.62±35.36
**400–500**	47.08±5.36	70.82±33.41	54.02±11.37	58.70±9.84	96.10±42.70	55.08±9.09	94.92±41.37

	All FS≤1 (21)	FS P = 2 (15)	FS P = 3 (25)	FS C = 2 (18)	FS C = 3 (31)	FS S = 2 (34)	FS S = 3 (15)
**T25FW** 2	3.80±0.57	4.62±1.10	6.62±2.93	4.24±0.83	6.40±2.69	4.74±1.59	7.08±3.37
**T25FW^+^** 3	3.48±0.67	4.20±0.91	5.78±1.94	3.89±0.73	5.66±1.80	4.30±1.20	6.12±2.26
**T100MW** 4	48.04±7.39	58.20±13.16	80.80±29.24	52.96±9.01	78.86±27.10	60.29±18.37	83.43±34.25
**T500MW** 5	254.86±29.69	320.97±76.14	456.92±173.69	291.83±60.60	446.01±158.83	336.86±115.79	467.63±196.46
**0–100**	48.48±7.43	61.81±16.02	83.03±25.99	55.27±10.71	81.75±24.14	62.85±19.98	85.54±28.51
**100–200**	51.37±4.96	65.48±16.90	88.72±32.74	57.68±11.63	88.13±29.79	67.03±23.19	92.11±35.75
**200–300**	52.07±5.85	64.63±14.48	90.64±35.27	58.98±11.35	88.41±32.22	67.11±21.49	94.18±40.53
**300–400**	51.62±5.93	64.33±14.94	94.76±38.06	59.98±13.44	91.90±34.79	68.54±23.09	96.59±44.14
**400–500**	51.33±7.11	64.71±15.34	99.77±45.38	59.92±14.34	95.82±41.79	71.33±31.39	99.21±48.64

The number of subjects in each subgroup is indicated in brackets next to subtitles.

1 : each time performance is expressed in seconds, as mean ± SD;

2 : Timed 25-Foot Walk Test;

3 : Corrected version of the T25FW with a dynamic start;

4 : Timed 100-Meter Walk Test;

5 : Timed 500-Meter Walk Test with lap times evaluated for every 100 meter interval.

**Figure 2:**
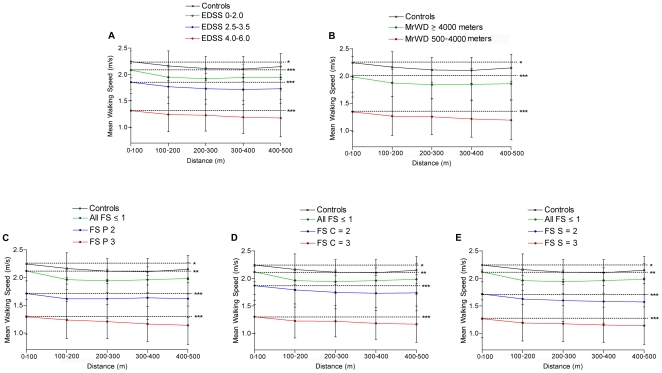
MWS over five successive 100 m interval laps along the T500MW. Subgroup analysis are presented in healthy volunteers and in different subgroups of the pMS population, stratified according to their EDSS (A), their maximum reported walking distance (MrWD) (B), and their pyramidal (C), cerebellar (D) and sensitive (E) functional scores (FS). The dashed lines represent the comparison between the “baseline” MWS of the first 100 m (T0–100MW) and the “final” MWS of the last 100 m (T400–500MW) for all subgroups. t-test values were *p<0.05, **p<0.01, ***p<0.0001.

In the T500MW, MWS was calculated over the five successive 100 m interval laps in order to capture the motor fatigue related deceleration occurring over time during this demanding motor task (Table 2, Figure 2). Different patterns of MWS evolution were observed in regard of the type of population studied (Figure 2). Regardless of the absolute differences of their MWS, healthy volunteers and pMS with a low level of disability (i.e. with an EDSS≤2.0, MrWD≥4000 m or all FS scores ≤1, Figure 2A, 2B and 2C, D, E, respectively) significantly decelerated during a 500 m walking task, as demonstrated by the comparison between the MWS of the first 100 m (T0–100MW) and the MWS of the last 100 m (T400–500MW) during the test (p = 0,0104 for healthy volunteers, p<0,0001 for pMS with MrWD≥4000 m and p = 0,0089 for pMS will all FS scores ≤1). A mild acceleration at the end of the task (i.e. a higher MWS during the last 100 m - T400–500 - compared to the MWS over the T300–400) was observed in healthy volunteers and pMS with all FS scores ≤1, but only reached significance in the healthy volunteers population (p = 0,0286, data not shown). A highly significant deceleration was consistently observed in more disabled pMS with an EDSS 2.5–3.5 and 4.0–6.0 (Figure 2A), a MrWD between 500 and 4000 m (Figure 2B) or Kurtzke FS scores at 2 or 3 in the pyramidal, cerebellar or sensitive systems (Figure 2C, 2D and 2E, respectively). For these latter more disabled pMS groups all p values were <0,0001 for the comparisons of MWS between T0–100MW and T400–500MW.

**Figure 3:**
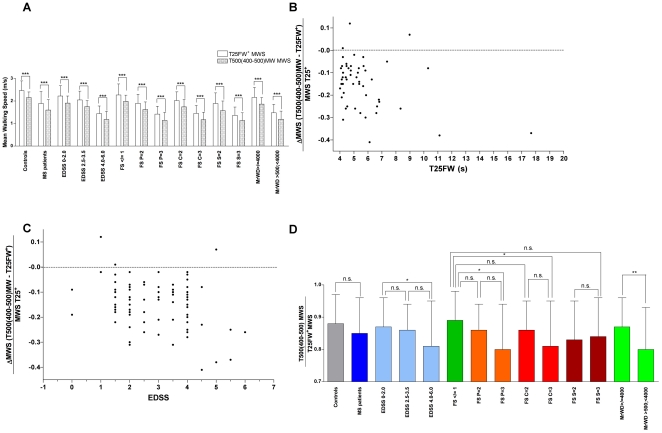
Quantification of ambulation fatigability through the Deceleration Index (DI). Ambulation fatigability was evaluated through the integration of the fastest (T25FW^+^) and the lowest (T400–500MW) measurable WS, which were obviously highly statistically different in all pMS subgroups and healthy volunteers (A, all p<0,0001). Absolute WS differences were however very similar between all groups (ranging from 0.22 m/s for FS S = 3 to 0.31 m/s for EDSS 2.5–3.5 and Controls). This is why further comparisons between groups were focused on relative WS changes. No significant correlation could be found between the individual values of relative deceleration evaluated by the difference of MWS on the T25FW^+^ and on the T400–500MW (expressed as percentage of the T25FW^+^ MWS) and the level of walking impairment according to the T25FW (B) or the EDSS status (C). Deceleration Index (DI) calculated as the ratio between MWS of the T400–500MW divided by MWS of the T25FW^+^ (D) in healthy volunteers and in different subgroups of the MS population.

In order to quantify ambulation fatigability over a demanding distance of effort, we proposed to integrate the fastest and the lowest measurable walking speeds over the different tested walking paradigms. The T25FW^+^ MWS was previously confirmed to be a valid test to approach the fastest MWS of MS patients on a very short distance regardless of their acceleration capacity [10]. On the other hand, the mean finishing pace during the last 100 m of the T500MW (T400–500MW) appeared to be the lowest measurable speed over this fatigue inducing longer distance (Figure 2). The difference between T25FW^+^ MWS and T400–500MW MWS was obviously significant in all pMS subgroups and healthy volunteers (Figure 3A, all p<0,0001). The individual performances of pMS showed that the relative deceleration observed between MWS values of the T25FW^+^ and T400–500MW (expressed as percentage of the T25FW^+^ MWS) was highly variable at all levels of walking impairment (stratified according to the T25FW, Figure 3B) and EDSS status (Figure 3C). We calculated the so-called Deceleration Index (DI) as the ratio between MWS of the T400–500MW divided by MWS of the T25FW^+^ (Figure 3D). Hence, the lower the DI ratio is, the more pronounced the patients were subjected to fatigue-related decrease of their walking pace over a long distance effort evaluated here by the 500 m dash. We observed a non significantly lower DI for pMS altogether compared to healthy controls (p = 0,088). pMS with an EDSS 4.0–6.0 had a significantly lower DI compared to pMS with an EDSS≤2.0 (p = 0.045). Compared to pMS with pyramidal, cerebellar and sensitive FS scores all ≤1, pMS with pyramidal or cerebellar FS at 2 had a non significantly lower DI (p = 0.33 and p = 0.42, respectively), whereas pMS with pyramidal or cerebellar FS at 3 had a significantly lower DI (p = 0.02 and p = 0.03, respectively). In contrast, pMS with a sensitive FS at 2 or 3 had a lower DI than pMS with all FS scores ≤1 but the differences were not significant for both comparisons. The DI of pMS subjects with a MrWD between 500 m and 4000 m was significantly lower than for pMS with a MrWD≥4000 m (p = 0.0044). Finally, in contrast to the differences measured over absolute walking performances in short or long distance walking tests, no significant differences were observed for DI values between healthy volunteers and pMS with a low level of disability (i.e. with an EDSS≤2.0, MrWD≥4000 m or all FS scores ≤1, statistics not graphically shown on Figure 3D).

## Discussion

This study evaluated the relative walking speed performances of pMS compared to healthy volunteers on short and long distance walking tests. The groups were well matched according to BMI and sex ratio but the higher age in the pMS population compared to healthy volunteers may have slightly influenced the observed differences since the mean WS probably decreases with age [20].

All walking tests were performed in the “as fast as you can” configuration of the task in order to downsize motivational interferences, which are probably more prominent in a “preferred pace” modality [21].

We demonstrated that in a cohort of pMS with mild to moderate disability and EDSS scores ranging up to 6.0, the evaluation of walking capacities over 500 m was an achievable goal, as long as assistive devices and short stops if needed were allowed for the more disabled patients between EDSS 4.5 and 6.0. The range of performances of our pMS population was globally in line with that of previous studies evaluating walking speed on similar distances [2], [12], [22].

The absolute performances of pMS obviously decreased according to the EDSS score, but a significant ambulation impairment was already seen on short and long distance in pMS with mild disability, with an EDSS status ≤2.0 or a MrWD≥4000 m [16], [23].

We observed various patterns of deceleration in the different subsets of pMS over a 500 m walking task, regardless of absolute timed performances. As previously described, healthy volunteers and pMS with minimal disability (all FS scores ≤1, i.e. EDSS≤1.5) retained the ability to accelerate during the last 100 m of the 500 m task [2], [12]. This final WS acceleration referred to the comparison between the T400–500 and the T300–400. However the mean WS of the T400–500 remained significantly lower than the mean WS of T0–100 for all subgroups. This observation is probably related to motivational issues (“end of the task” phenomenon), but it is striking that no final WS acceleration was observed in more disabled pMS, which may reflect the consequences of a more severe cognitive impairment or the translation of an increased spasticity or both aspects. For pMS with significant disability ranging from EDSS 2.0 to 6.0, the finishing pace of the last 100 m of the T500MW was the slowest measurable WS across the 4 walking tests. In contrast, the mean WS on T25FW^+^ with a propelled start provided the fastest measurable WS in all pMS subgroups.

In order to assess locomotor fatigue, we identified the deceleration index (DI) as a ratio between the minimal (T400–500) and maximal (T25FW^+^) measurable WS. The origin of walking fatigability was not investigated in the current study, but it is noteworthy that pMS with a value of 3 on pyramidal or cerebellar FS scores demonstrated a significant alteration in the DI whereas pMS with a value of 3 on sensitive FS score did not. The individual DI of pMS were highly variable at all stages of walking impairment and the mean DI was significantly lower only in pMS with EDSS 4.0–6.0 or a maximum reported walking distance ≤4000 m. The mean DI remained similar to healthy volunteers in pMS with a low level of disability (i.e. with an EDSS≤2.0, MrWD≥4000 m) while absolute walking performances on short or long distance walking tests were all significantly abnormal in these pMS subgroups at early disease stages.

These results indicate that the DI measures the alteration of a sustained performance throughout a long demanding walking task, which is not captured by conventional absolute WS measurements, whether on a specific short or long distance, or in time-based settings. Such findings are consistent with the previous demonstration that motor fatigue is partially independent from motor (pyramidal) weakness [2], [24].

In regard of the usual 500 m MrWD delineated by the EDSS calculation rules, this work suggested that a MrWD of 4000 m may be a more reliable threshold to better discriminate between “fully ambulatory” (as termed by John F. Kurtzke) and significantly limited pMS according to their walking performances. Although the 4000 m was chosen arbitrarily, a higher threshold may have led to consider healthy untrained individuals as disabled.

It was outside the scope of the present cross-sectional analysis to investigate the sensitivity to change of the walking tests and their relevance in self-reported quality of life of pMS but it will be prospectively addressed in a future study.

In conclusion, we provided evidence that sequential gait evaluation over a 500 m distance is a valuable tool to measure the decrease of WS over the duration of a demanding walking task. The combination of short and long distance “as fast as possible” walking tests to assess a relative deceleration (DI) is a coherent paradigm to allow a reliable measurement of locomotor fatigue. Our data suggest that ambulation fatigability is at least partially independent from absolute performances on a given distance, which are abnormal early in MS, while the DI is altered later in the disease course. The DI may be a sensitive tool to detect and measure walking fatigability even though it is less sensitive than absolute mean WS on short and long distances to detect early walking impairment. Further work will be needed to clarify the clinical relevance of such a new performance-based measurement.

## Supporting Information

Table S1 Statistical comparisons of the MWS (Student T-tests) in the T25FW, the T25FW^+^, the T100MW and the T500MW across different subsets of the pMS population stratified according to their pyramidal (P), cerebellar (C) and sensitive (S) Functional System Scores (FS).
(DOC)
