# Supplementary material for: Motor Fatigue Measurement by Distance-Induced Slow Down of Walking Speed in Multiple Sclerosis
Source: PLoS One. 2012 Apr 13;7(4):e34744. doi: 10.1371/journal.pone.0034744 (PMC3326046; doi:10.1371/journal.pone.0034744)
Supplement: Table S1 — Statistical comparisons of the MWS (Student T-tests) in the T25FW, the T25FW+, the T100MW and the T500MW across different subsets of the pMS population stratified according to their pyramidal (P), cerebellar (C) and sensitive (S) Functional System Scores (FS). (DOC) [file pone.0034744.s001.doc]

**Supplemental table S1 : Statistical comparisons of the MWS (Student T-tests) in the T25FW, the T25FW^+^, the T100MW and the T500MW across different subsets of the pMS population stratified according to their pyramidal (P), cerebellar (C) and sensitive (S) Functional System Scores (FS)**

|  | FS P |  | FS C |  | FS S |  |
| --- | --- | --- | --- | --- | --- | --- |
|  | 2 | 3 | 2 | 3 | 2 | 3 |
| T25FW | | | | | | |
| All FS≤1 | 0.0141* | n.d. | 0.1036 | n.d. | 0.0075** | n.d. |
| P2 | n.d. | 0.0009*** | n.d. | n.d. | n.d. | n.d. |
| C2 | n.d. | n.d. | n.d. | P<0.0001*** | n.d. | n.d. |
| S2 | n.d. | n.d. | n.d. | n.d. | n.d. | 0.0003*** |
| T25FW^+^ | | | | | | |
| All FS≤1 | 0.0198* | n.d. | 0.0911 | n.d. | 0.0057** | n.d. |
| P2 | n.d. | 0.0004*** | n.d. | n.d. | n.d. | n.d. |
| C2 | n.d. | n.d. | n.d. | P<0.0001*** | n.d. | n.d. |
| S2 | n.d. | n.d. | n.d. | n.d. | n.d. | 0.0003*** |
| T100MW | | | | | | |
| All FS≤1 | 0.0137* | n.d. | 0.0918 | n.d. | 0.0037** | n.d. |
| P2 | n.d. | 0.0004*** | n.d. | n.d. | n.d. | n.d. |
| C2 | n.d. | n.d. | n.d. | P<0.0001*** | n.d. | n.d. |
| S2 | n.d. | n.d. | n.d. | n.d. | n.d. | 0.0015** |
| T500MW | | | | | | |
| All FS≤1 | 0.0012** | n.d. | 0.0211* | n.d. | 0.0004 | n.d. |
| P2 | n.d. | 0.0003*** | n.d. | n.d. | n.d. | n.d. |
| C2 | n.d. | n.d. | n.d. | P<0.0001*** | n.d. | n.d. |
| S2 | n.d. | n.d. | n.d. | n.d. | n.d. | 0.0009*** |

*=p<0.05

**=p<0.01

***=p<0.001

n.d.= not determined
